# Assessing the impact of sex and circadian rhythms on rodent behavior: refining preclinical study designs

**DOI:** 10.31744/einstein_journal/2025AO1614

**Published:** 2025-10-13

**Authors:** Olívia Furiama Metropolo Dias, Nicole Mastandrea Ennes do Valle, Fernando Anselmo de Oliveira, Arielly da Hora Alves, Javier Bustamante Mamami, Gabriel Nery de Albuquerque Rego, Keithy Felix da Silva, Marta Caetano dos Santos Galanciak, Juan Matheus Munoz, Luciana Cintra, Mariana Penteado Nucci, Lionel Fernel Gamarra

**Affiliations:** 1 Hospital Israelita Albert Einstein São Paulo SP Brazil Hospital Israelita Albert Einstein, São Paulo, SP, Brazil.; 2 Universidade de São Paulo LIM44 - Hospital das Clínicas Faculdade Medicina São Paulo SP Brazil LIM44 - Hospital das Clínicas, Faculdade Medicina, Universidade de São Paulo, São Paulo, SP, Brazil.

**Keywords:** Behavior, animal, Circadian rhythm, Sex, Locomotion, Cognition, Spatial memory, Memory, episodic, Neurodegenerative diseases, Rats, Wistar

## Abstract

**Objective:**

This study aimed to investigate the effects of sex and time of day on behavioral parameters in healthy rats, focusing on locomotion, cognition, and memory (both spatial and episodic).

**Methods:**

Twenty-four Wistar rats (12 males and 12 females) were divided into morning and afternoon groups. Behavioral tests included actimetry for spontaneous locomotion, novel object recognition test for episodic memory, and the Morris water maze test for spatial memory. Data were analyzed cross-sectionally and longitudinally, considering sex and time of day as variables. The evaluated parameters included fast and slow horizontal and vertical movements, recognition index, average speed, and quadrant preference in the Morris water maze.

**Results:**

Females exhibited higher frequencies of rapid horizontal and vertical movements than males, especially in the morning. Novel object recognition test results showed higher recognition index values in the morning, with females displaying greater exploration of novel objects, while the Morris water maze test results indicated that the time spent in the target quadrant was consistent across groups, but females demonstrated longer latencies in the afternoon.

**Conclusion:**

These results highlight the significant influence of sex and circadian timing on behavioral performance in healthy animals. These factors should be carefully considered when designing and interpreting preclinical behavioral studies to improve experimental consistency and data reproducibility.

## Highlights

■Sex plays a crucial role in behavioral assessments of preclinical study designs.

■Circadian rhythm influences episodic memory performance in rodent behavior.

■Spatial memory exhibits fewer variations than locomotion and episodic memory.

■Sex and the circadian cycle are critical factors in behavioral assessment.

## In Brief

This study showed that biological sex and circadian rhythm significantly influenced behavioral tests commonly used in preclinical models of neurodegenerative and psychiatric disorders. Female rats exhibited higher locomotor activity than males, especially during the afternoon. Episodic memory was better in the morning in both sexes, while spatial memory showed minor circadian effects.

## Graphical abstract

**Figure:**
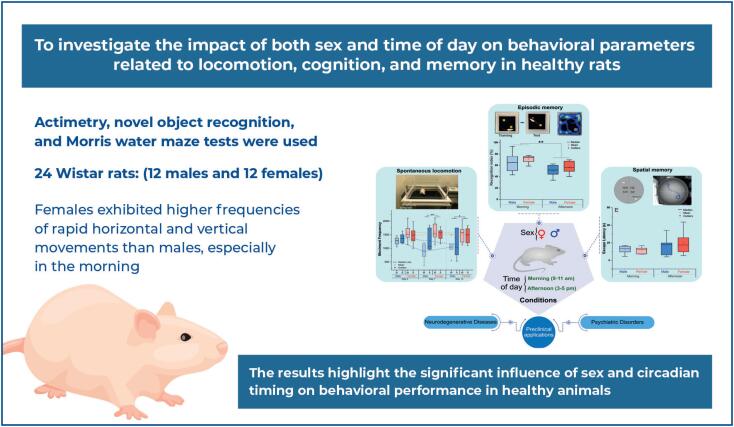

## INTRODUCTION

Understanding how biological variables, such as sex and circadian rhythms, influence behavior is critical for improving the translational relevance of preclinical research. Although rodent models are widely used to investigate mechanisms underlying human physiology and disease, experimental designs often overlook these fundamental factors. This oversight may have contributed to inconsistencies and limited reproducibility across studies. Given the growing emphasis on precision medicine and personalized health approaches in the human population, refining preclinical protocols to account for sex differences and time-of-day effects is essential.^( 1 , 2 )^ These variables also play a critical role in behavioral studies targeting neurological and psychiatric disorders, where standardized tests aim to assess motor, cognitive, and affective functions in animal models.^( 3 )^

Neurodegenerative disorders, such as Alzheimer's disease, Parkinson's disease, amyotrophic lateral sclerosis, and Huntington's disease, represent a significant global health challenge due to their progressive nature and the absence of effective treatments,^( 4 , 5 )^ and aging is the main risk factor for most of these diseases.^( 6 )^ These conditions are characterized by the gradual loss of neuronal function and structure, leading to cognitive and motor deficits that severely compromise patients’ quality of life.^( 7 )^ Psychiatric disorders, such as major depression, anxiety disorders, and schizophrenia, have also been extensively investigated using behavioral tests in animal models, with the goal of elucidating their neurobiological basis and evaluating therapeutic interventions.^( 8 - 12 )^ Although the development of therapeutic strategies for these diseases often relies on behavioral observations in animal models, the direct relevance and generalizability of basic behavioral data obtained from healthy animals, such as those from the present study, remain to be established. Therefore, caution should be exercised when extrapolating such findings to other pathological conditions.

Behavioral assessment is an essential tool for studying animal models of neurological and psychiatric disorders, allowing the investigation of motor function, cognitive ability, and memory (spatial and episodic).^( 13 )^ Tests, such as the novel object recognition, Morris water maze, and actimetry, provide critical data for evaluating potential therapeutic interventions. However, the interpretation and reproducibility of behavioral data can be influenced by various experimental factors, including circadian timing and biological sex, which are frequently underreported or uncontrolled in study designs.^( 14 , 15 )^

Circadian rhythms are known to influence functions, such as locomotion, memory, and learning, and sex-specific hormonal variations can further modulate these behaviors. If not properly controlled, these factors can introduce bias or variability, hindering the translation of preclinical findings into clinical practice.^( 16 , 17 )^ In particular, sex-related behavioral differences, especially in locomotor and exploratory activity, may not only reflect motor capacity, but also aspects, such as motivation, curiosity, and cognitive engagement. Recognizing and characterizing these patterns are important for a more accurate interpretation of behavioral test results.^( 18 )^

In this context, the present study aimed to investigate the impact of sex and time of day (morning vs. afternoon) on behavioral parameters related to locomotion, cognition, and memory (spatial and episodic) in healthy rats. Using actimetry, novel object recognition, and Morris water maze tests, groups were compared by time and sex to identify potential behavioral patterns. The findings will inform the design of future behavioral experiments by highlighting the influence of biological sex and circadian timing on experimental variability, thereby supporting the refinement of experimental protocols in basic and preclinical research.

## OBJECTIVE

To investigate the effects of sex and time of day on behavioral parameters in healthy rats, focusing on locomotion, cognition, and memory (both spatial and episodic).

## METHODS

### Ethics statement and animals

This study was approved by the Ethics in Animal Research Committee of *Hospital Israelita Albert Einstein* (São Paulo, Brazil; approval number: 4850/21). Twelve 3-month-old female (weight 209±20g) and twelve 3-month-old male (weight 340±50g) Wistar rats were used. The animals were maintained at 21±2°C and 60%±5% relative humidity, with full ventilation, under a 12 h light/dark cycle (7:00 AM –7:00 PM), and had access to food and water ad libitum at *Centro de Experimentação e Treinamento em Cirurgia* , a vivarium accredited by the Association for the Assessment and Accreditation of Laboratory Animal Care International.

### Experimental design

**Figure 1:**
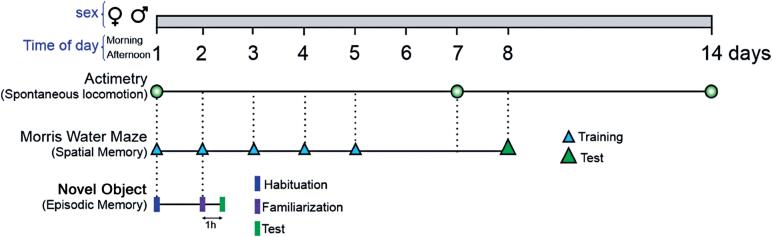
Representative timeline of the experiments. The first is the general timeline (from 1 to 14 days). The second is the actimetry test timeline, with green circles indicating the date of application of the text (1^st^, 7^th^, and 14^th^). The third is the Morris water maze timeline, with blue triangles representing the training phase (from days 1 to 5) and green indicating the test phase (8^th^ day). The fourth is the novel object test timeline, with blue bars representing the habituation phase (1^st^ day), pink bars indicating the familiarization phase (2^nd^ day), and green bars representing the test phase (2^nd^ day, after 1 h of familiarization). The experiments were conducted with both male and female subjects and evaluated at two different times of day: morning and afternoon

The timeline of all experiments is shown in figure 1 . Comparisons were made between sessions (over 14 days). The animals were divided randomly into groups based on the time of day for training in the morning (between 9:00 AM and 11:00 AM) and afternoon (between 3:00 PM and 6:00 PM). The time of day for grouping was maintained in each group of males (n=6) and females (n=6) for sex effect evaluation. The groups were named as follows: F_m (Female in the morning), F_a (Female in the afternoon), M_m (Male in the morning), and M_a (Male in the afternoon).

All indoor experiments were conducted at a constant temperature of 22±2ºC, with controlled humidity of 40–60%. One researcher was assigned to each periodic group to oversee all habituations and experiments, reducing any possible manipulation bias.

All the animals were habituated to the experimental room 30 min before each behavioral test. To eliminate olfactory cues, all equipment and the arena were sanitized with 5% alcohol between animals, and when necessary, between sessions.

### Spontaneous locomotion assessment by actimetry

Spontaneous locomotor activity was analyzed using the infrared Actimeter LE 8825 system (Actitrack, PanLab Harvard Apparatus, Barcelona, Spain). The equipment comprised a two-dimensional (X- and Y-axes) square frame of 70×70cm² surrounded by 30cm high transparent walls, frame support, and control unit. Each animal was placed in the center of the arena and tracked for 5 min.

The experiment was repeated once a week for 3 weeks (D0, D7, and D14). No training was required. During the experiment, the researcher remained outside the room to avoid interference. The four movement parameters used for comparison between groups and sessions were slow horizontal (S-MOV), fast horizontal (F-MOV), slow rearing (S-REA), and fast rearing (F-REA). The rearing movement can also be considered vertical movement. Data were processed using SEDACOM v2.0.

For data analysis, horizontal and vertical movements (slow or fast) were analyzed separately, verifying the results by intersession data (longitudinal) and groups (transversal). Stereotyped movements were not considered in this analysis.

### Episodic memory assessment by novel object recognition test

The novel object recognition test evaluates an animal's ability to differentiate between novel and familiar objects. The test was conducted in an opaque acrylic arena measuring 70×70cm², with walls 30cm high, and a dark bottom. The experiment consisted of three stages: habituation, familiarization, and final test. During the habituation phase, animals were allowed to explore the arena freely for 10 min. After 24 h, the familiarization phase involved exposing the animals to two identical objects (10cm yellow cones) for 5 min. In the final test, conducted 1 h after familiarization, one of the cones was replaced with a red block of the same height, and the animals were allowed to explore for 3 min. This phase was recorded and analyzed using EthoVision XT software (Noldus Information Technology, Netherlands). The experiment was performed only once for each animal, and all tests were conducted under dim lighting conditions (20–40 lux) to ensure recording quality.

The parameters obtained using EthoVision XT were velocity, exploration time of the familiar object (T_F_), and exploration time of the new object (T_N_). The actions of touching, smelling, and approaching an object within 2 cm were considered for exploration. For data analysis, the recognition index (RI) was expressed as *T*_N_ *x100* /( *T*_N_ *+T*_F_).

### Spatial memory assessment by Morris water maze test

The Morris water maze test assesses spatial learning and memory using a circular pool with a diameter of 180cm and a platform with a diameter of 10cm. Training was conducted over four consecutive days, with three trials per day. During the training, the platform was visible and marked with a visual cue (yellow flag). Each trial involved placing the animal in the pool and allowing it to search for the platform for up to 1 min or until the platform was found. Seventy-two hours after completing the training, the final test was conducted in a single 1-min trial with the platform hidden and no visual cues. Only the final test was recorded and analyzed using EthoVision XT software (Noldus Information Technology, Netherlands).

The pool was virtually divided into four quadrants (southeast [SE], southwest [SW], northeast [NE], and northwest [NW]), with the platform consistently positioned in the SE quadrant throughout all stages. The animals were released into the pool from the NW quadrant. The water temperature was maintained at 33±3 °C using a thermostat, and 300 g of powdered milk was added to the water to obscure the pool bottom and eliminate visual cues. To ensure proper video recording, the tests were conducted under dim lighting conditions (20–40 lux). The experiment, comprising two stages (training and final tests), was performed once for each animal.

The parameters obtained using EthoVision XT were escape latency, average speed (path length/escape latency), and time spent per quadrant. For data analysis, only a transversal assessment was performed, and all animals that did not find the platform within the total trial time were excluded from the final statistical analysis.

### Statistical analysis

Data are presented as mean and standard deviation. Inferential statistical analysis was performed using the two-way and repeated measures analysis of variance (ANOVA), followed by post-hoc test corrected by Tukey's test using JASP software version 0.18.1 (https://jasp-stats.org/; accessed on 14^th^ July 2023). Statistical significance level was set at p<0.05.

## RESULTS

### Animal characteristics

Throughout the experimental period, the animals were closely monitored for behavioral changes, appetite, water consumption, and social interactions. No signs of isolated behavioral changes, such as stress or barbering, were observed. Food intake and water consumption were normal. The animals did not show signs of isolation and consistently interacted with at least two other rodents per cage. Body weight showed no significant changes during the experiment, with an average weight gain of 17g in males and 7g in females, indicating stable health and wellbeing.

### Actimetry

**Figure 2:**
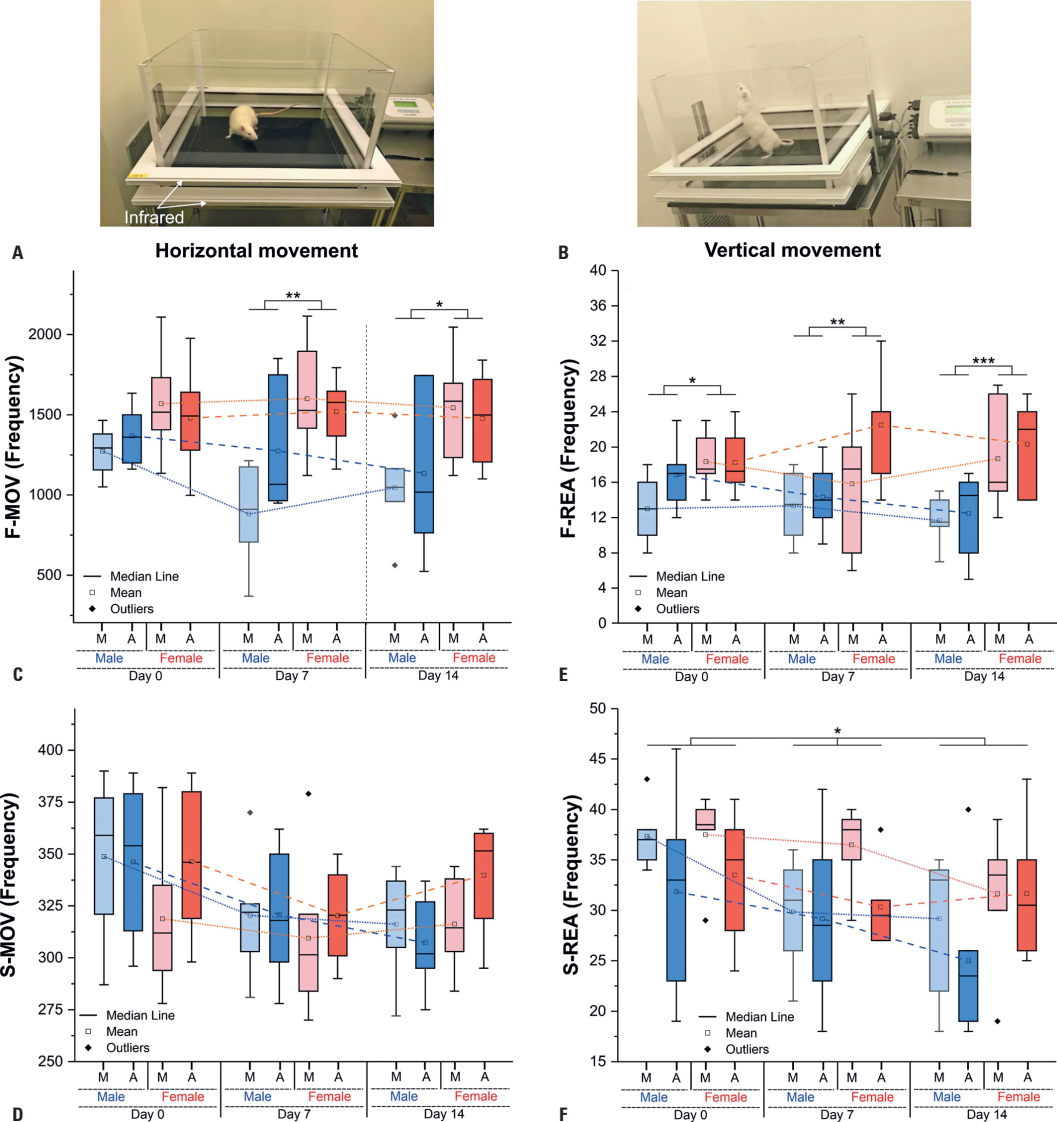
Spontaneous movement was assessed by actimetry, analyzing horizontal movement (A) and vertical movement (B). Horizontal movement was further classified into fast (C) and slow (D) patterns, while vertical movement was divided into fast (E) and slow (F) patterns. Males in the morning are represented in light blue, males in the afternoon in dark blue, females in the morning in light red, and females in the afternoon in dark red

Spontaneous movements were evaluated based on the frequencies of horizontal and vertical movements at both fast and slow frequencies ( Figure 2A and B ). This analysis considered variables, such as sex and time of day (morning and afternoon), in each session (0, 7, and 14 days) or between sessions (longitudinal).

*p<0.05; **p<0.01, and ***p<0.001.

F-MOV: fast horizontal movement; F-REA: fast vertical movement; S-MOV: slow horizontal movement; S-REA: slow vertical movement; M: morning; A: afternoon.

On day 0 (D0), cross-sectional analysis of F-MOV, considering the effects of sex, time of day, and their interaction, revealed no significant differences across factors ( Table 1 ). The groups showed similar movement patterns, with slightly higher values observed in females in both the morning and afternoon. In contrast, on days 7 (D7) and 14 (D14), a significant effect of sex was observed (p=0.002 and p=0.012, respectively), with females demonstrating greater movement than males, while the effects of time of day and their interaction were not significant ( Figure 2B and C ; Table 1 ). The post-hoc test conducted on D7 and D14 identified a significant difference only on D7, specifically between sexes at different times (comparing F-a with M-m; p=0.017) and between sexes in the morning (p=0.007).

**Table 1:** Descriptive and ANOVA analyses of the movement frequency in each parameter of the spontaneous movement test, considering fast and slow horizontal movement (F-MOV and S-MOV) and vertical movement (F-REA and S-REA) by sex (female and male), time of day (morning and afternoon), and session (days 0, 7, and 14)

Session	Condition	Female		Male		Anova (p value)		
	F-MOV	Morning	Afternoon	Morning	Afternoon	Sex effect	Time effect	Interaction
D0	Mean	1569.2	1480.0	1272.8	1369.2	0.073	0.974	0.399
	SD	334.6	332.4	154.1	178.5			
D7	Mean	1600.5	1520.8	881.0	1274.5	0.002 **	0.265	0.099
	SD	359.9	223.9	313.5	414.8			
D14	Mean	1544.5	1477.5	1046.0	1135.5	0.012 *	0.942	0.613
	SD	334.5	307.3	302.7	509.5			
ANOVA (p-value)	Session effect	0.961	0.959	0.067	0.596			
	S-MOV							
D0	Mean	318.8	346.3	348.8	346.2	0.356	0.441	0.352
	SD	36.5	35.1	39.1	40.5			
D7	Mean	309.5	320.3	320.4	320.6	0.682	0.685	0.700
	SD	23.5	23.5	32.9	31.5			
D14	Mean	316.3	339.8	316.2	307.2	0.159	0.524	0.162
	SD	23.4	26.9	28.8	24.9			
ANOVA (p-value)	Session effect	0.885	0.299	0.259	0.200			
	F-REA							
D0	Mean	18.2	18.3	16.8	13.0	0.034 *	0.221	0.202
	SD	3.6	3.2	3.8	3.9			
D7	Mean	15.8	22.5	13.3	14.3	0.033 **	0.115	0.238
	SD	7.6	6.3	4.2	3.9			
D14	Mean	18.7	20.3	11.7	12.5	0.001 ***	0.542	0.838
	SD	6.3	5.2	2.8	4.8			
ANOVA (p-value)	Session effect	0.676	0.384	0.713	0.232			
	S-REA							
D0	Mean	37.5	33.5	37.3	31.8	0.730	0.085	0.775
	SD	4.3	6.4	3.2	9.7			
D7	Mean	36.5	30.3	29.8	29.2	0.118	0.170	0.265
	SD	4.1	4.1	5.6	8.6			
D14	Mean	31.7	31.7	29.2	25.0	0.136	0.488	0.488
	SD	6.8	6.8	7.2	7.9			
ANOVA (p-value)	Session effect	0.153	0.653	0.043 *	0.419			

* p<0.05;

** p<0.01,

*** p<0.001.

F-MOV: fast horizontal movement; S-MOV: slow horizontal movement; F-REA: fast vertical movement; S-REA: slow vertical movement; D0: Day 0; D7: Day 7; D14: Day 14; SD: standard deviation.

Longitudinal analysis of F-MOV within each group ( Figure 2C ) showed that the movement patterns of females consistently remained higher over time than those of males. However, the ANOVA test showed no significant session effect ( Table 1 ).

For S-MOV ( Figure 2D ), the cross-sectional analysis revealed similar behavior in both sexes and time of day on D0, D7, and D14, with no significant differences in movement frequency for these variables, as determined by ANOVA ( Table 1 ). Similarly, in the longitudinal analysis of S-MOV within each group, ANOVA indicated no significant session effects (D0, D7, or D14) for any group ( Table 1 ). Despite the lack of significant results in both cross-sectional and longitudinal analyses, female afternoon movements consistently showed a higher frequency than male afternoon movements over time. In contrast, female morning movements had a lower frequency than male morning movements on days 0 and 7; however, by day 14, females had outperformed males in both the morning and afternoon sessions, similar to the observations for F-MOV.

In cross-sectional analysis for F-REA ( Figure 2E , Table 1 ), significant differences were observed only for the sex effect in all sessions (D0, p=0.034; D7, p=0.033; and D14, p=0.001), with females maintaining a higher frequency than males in all sessions. The post-hoc analysis indicated significant differences between male morning (M_m) and female afternoon (F_a) on D14 (p=0.030), and a trend of significance was observed for the same comparison on D7 (p=0.052) between F_a and M_a on D14 (F_a: p=0.055).

Longitudinally, females exhibited a higher frequency of vertical movements than males, a pattern observed in rapid horizontal movements. However, this increase in females was more pronounced in vertical movements during the afternoon, as was also evident in males. Despite these differences, no significant effects were observed across sessions in any group, as shown in figure 2 E and table 1 .

Cross-sectional analysis of S-REA ( Figure 2F ) demonstrated that females had higher movement frequency than males at all time points. Interestingly, both sexes exhibited higher movement frequencies in the morning than in the afternoon, mirroring the pattern observed for F-MOV. However, ANOVA revealed no significant differences in any session (D0, D7, or D14). In the longitudinal analysis, only M_m showed significant results by ANOVA (p=0.043), with the post-hoc test indicating a marginal difference between D0 and D14 (p=0.058), suggesting a sharp decline in movement frequency over time.

### Novel object recognition test

**Figure 3:**
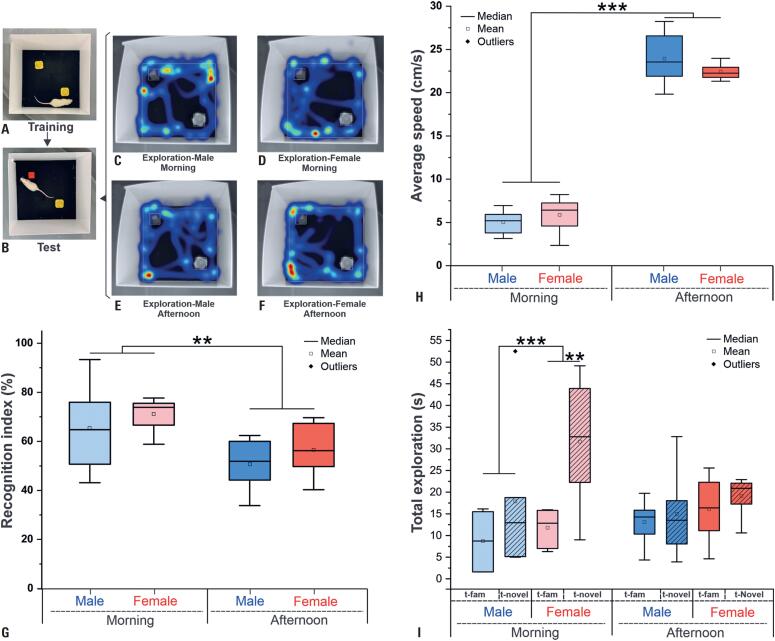
Behavioral assessment by the novel object recognition test by time of day and sex. (A and B) Training and test phases, respectively, in which the animals were exposed to two identical objects to familiarize themselves with the environment, then one object was replaced with a novel object, and the exploration of both objects was measured. (C-F) Heatmaps illustrating exploration patterns for male and female animals during the morning (C, D) and afternoon (E, F) periods, showing the distribution of activity across the test arena. (G) Recognition index (RI, %). (H) Average speed (cm/s). (I) Total exploration time (s) for the familiar (t-fam) and novel (t-novel) objects. Blue represents males, and red represents females, with lighter shades indicating data collected during the morning and darker shades representing data from the afternoon

In the novel object recognition test ( Figure 3A – F ) by RI among sex, time of day, and their interaction ( Figure 3G and Table 2 ), a significant difference was only observed for the time effect (ANOVA: morning × afternoon, p=0.012), with a higher RI in the morning for both sexes. However, the post-hoc analysis only showed a trend (p=0.058) in the comparison between females in the morning and males in the afternoon, with extreme results between the variables.

**Table 2:** Descriptive and ANOVA analysis of the novel object recognition test on recognition index, total exploration time, average speed by sex (female and male), and time of day (morning and afternoon)

Parameter	Female		Male		Sex effect	Anova (p-value)		
	Morning	Afternoon	Morning	Afternoon		Time effect	Type of exploration	Interaction
Recognition Index								
Mean	0.7	0.6	0.6	0.5	0.290	0.012 **	-	0.975
SD	0.10	0.1	0.2	0.1				
Speed								
Mean	5.9	22.4	5.0	23.9	0.694	<0.001 ***	-	0.178
SD	2.1	0.9	1.4	3.1				
Novel exploration								
Mean	31.6	19.1	17.9	14.9	0.050	0.571	0.006 **	0.412 @ 0.319 # 0.048 & * 0.425 £
SD	15.5	4.6	17.8	10.1				
Familiar exploration								
Mean	11.8	16.1	8.7	13.1				
SD	4.3	7.7	7.1	5.3				

* p<0.05;

** p<0.01,

*** p<0.001;

@ sex and time interaction;

# sex and type of exploration interaction;

& time and type of exploration interaction; and

£ sex, time, and type of exploration interaction.

SD: sandard deviation.

Analysis of the average speed for object exploration revealed a significant effect only at the time of the test (ANOVA: p<0.001), in which both sexes showed higher speed in the afternoon ( Figure 3H ; Table 2 ), which was confirmed by post-hoc analysis, and a significant difference (p<0.001) was observed between females in the morning and afternoon; the same was observed between males as well as between F-m and M-a and M-m and F-a.

*p<0.05; **p<0.01, and ***p<0.001.

In the analysis of total exploration between familiar and novel objects ( Figure 3I , Table 2 ), sex, and time, a significant result by ANOVA was observed for the type-exploring effect (familiar versus novel exploration: p=0.006), interaction between period and exploration type (p=0.048), and a trend for sex (p=0.050). Post-hoc analysis of the interaction of the three factors (type of exploration, time, and sex) indicated a significant difference only between the types of exploration (familiar versus new) for females in the morning (p=0.033, light red boxplot in Figure 3 I) and between the types of exploration (familiar versus new) and sexes in the morning (p=0.008, light red and blue boxplots in Figure 3 I). Therefore, as expected, the new object was explored more than the familiar object, and this exploration was more evident in females in the morning, with time of day not having much influence in males.

### Morris water maze test

**Figure 4:**
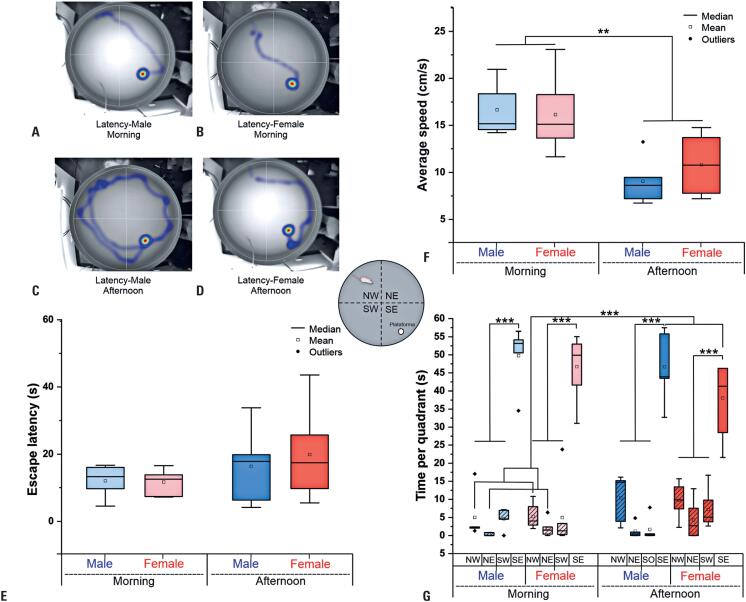
Morris water maze test performance by sex and time of day. (A-D) Representative heatmaps of latency to escape for male and female animals during the morning (A, B) and afternoon (C, D) sessions. These maps illustrate the swimming paths taken to locate the hidden platform. (E) Escape latency (s). (F) Average swimming speed (cm/s). (G) Time spent in each quadrant (s), labeled as NW (northwest), NE (northeast), SW (southwest), and SE (southeast, target quadrant). Blue represents males, and red represents females, with lighter shades for morning sessions and darker shades for afternoon sessions

Measurement of latency to reach the platform during the Morris water maze test ( Figure 4 A-E ) did not show significant differences when comparing sex, time of day, and the interaction of these factors ( Figure 4E and Table 3 ).

Average speed analyses ( Figure 4F and Table 3 ) showed a significant difference in only the effect of the evaluation period (morning greater than afternoon) (ANOVA, p=0.018), as also observed in the analysis of the new object, but in the opposite pattern (afternoon greater than morning); however, there was no significant difference in the two-by-two comparisons by post-hoc analysis of the time effect, with only a trend found between morning and afternoon in males (p=0.059).

**Table 3:** Descriptive and ANOVA analyses of the Morris water maze test of escape latency, time of exploration per quadrant, average speed by sex (female and male), and time of day (morning and afternoon)

Parameter		Female		Male		Sex effect	Anova (p-value)		
		Morning	Afternoon	Morning	Afternoon		Time effect	Quadrants effect	Interaction
Escape latency	Mean	11.7	19.9	12.1	16.4	0.709	0.145	-	0.644
	SD	3.7	13.6	5.0	11.9				
Speed	Mean	16.2	13.9	16.7	9.1	0.256	0.018 **	-	0.167
	SD	4.0	6.4	2.9	2.6				
SE	Mean	46.7	38.0	49.8	46.7	0.895	0.965	<.001 ***	0.998 @ 0.115 # 0.058 & * 0.593 £
	SD	9.0	10.8	8.8	10.2				
NW	Mean	5.3	9.7	5.0	10.4				
	SD	3.4	4.8	6.7	6.8				
NE	Mean	2.0	4.3	0.3	1.2				
	SD	2.3	5.3	0.4	2.1				
SW	Mean	5.0	7.2	4.6	1.7				
	SD	9.3	5.2	2.9	3.4				

* p<0.05,

** p<0.01,

*** p<0.001.

SE: southeast; NW: northwest; NE: northeast; SW: southwest; SD: standard deviation;

@ sex and time interaction,

# sex and quadrant interaction,

& time and quadrant interaction, and

£ sex, time, and quadrant interaction.

Analysis of the time of exploration per quadrant during the test ( Figure 4G and Table 3 ), sex, and period of test revealed a significant difference between quadrants (ANOVA: p<0.001); specifically, time of exploration, as well as the platform-placed target, was significantly greater in the SE than in the others (SW, NE, and NW; post-hoc test, p<0.001); moreover, it was significantly greater in the NW quadrant than in the NE quadrant (post-hoc test, p=0.029; Figure 4 G). The interaction between quadrants and period of test showed a trend of significance (p=0.058), and post-hoc analysis revealed a significant difference between SE morning and the morning and afternoon of other quadrants (p<0.001), between NW morning and SE afternoon (p<0.001), between NE morning and SE afternoon (p<0.001) and NW afternoon (p=0.043), between SO morning and SE afternoon, and between SE and NW, NE, and SW afternoon (p<0.001).

*p<0.05; **p<0.01, and ***p<0.001.

## DISCUSSION

This study demonstrated that animal behavior is influenced by both sex and circadian rhythms, even in the absence of any expressed pathology in animal models. For the main tests and models commonly used in the literature, these findings are particularly relevant for establishing protocols and experimental designs that are less affected by these variables, thereby ensuring more reliable and consistent results.

The approach used herein for evaluating spontaneous locomotion was more detailed than the traditional approach for assessing total locomotion in the open field test.^( 12 , 19 , 20 )^ Specifically, both horizontal and vertical movements, including their speed variations (slow and fast), were evaluated. Overall, significant differences between sexes were observed only during rapid movements (horizontal and vertical). For rapid horizontal movements, these differences were significant on days 7 and 14, while for rapid vertical movements, they were observed across all sessions, with females consistently showing a higher predominance of movements than males.

Kokras et al. also demonstrated that adult female Wistar rats exhibited higher frequencies of horizontal and vertical movements in both control states and after vehicle administration, as well as under experimental conditions involving the use of anxiolytics and antidepressants.^( 21 )^ Further, in an evaluation of exploratory locomotion prior to memory testing, adult Long Evans female rats were significantly more active and faster than males,^( 22 )^ supporting our findings. Another study evaluating the effects of biological sex, housing conditions, and time of day on social motivation found that female rats obtained more social rewards and exhibited greater activity, shorter latencies to initiate actions, and more locomotion than male rats.^( 2 )^

Although male animals have historically been favored in behavioral research to minimize the variability linked to the estrous cycle, this approach has led to sex-biased data that may compromise translational relevance.^( 11 )^ Recent discussions emphasize that relying exclusively on male subjects can obscure important biological differences, thereby reducing the generalizability and reproducibility of findings. Therefore, incorporating female animals into preclinical research is essential to ensuring more accurate, representative, and valid outcomes. The divergence observed between sexes likely results from a complex interplay of genetic, hormonal, and environmental factors, which should be systematically accounted for in experimental designs.^( 3 )^

Another interesting aspect evaluated, although not statistically significant, was the influence of the testing period (morning or afternoon) on rapid horizontal movements, in which the degree of the difference in movement frequency was much more pronounced. Across all sessions analyzed, females showed higher activity in the morning, while males displayed greater activity in the afternoon. This pattern can be attributed to the influence of hormones, such as estrogen, which are known to modulate locomotor activity in rodents, particularly during phases of heightened circadian alertness.^( 11 )^ This observation aligns with the findings of other studies, such as those by Marczinski et al. and Apelqvist et al.^( 1 , 23 )^ who also reported increased locomotor activity in females during specific circadian cycle periods. This heightened activity is likely influenced by hormonal and metabolic differences, particularly fluctuating levels of corticosterone and estrogen.^( 24 )^ Similar behavioral patterns have been observed in adult C57BL/6 mice, in which females exhibited greater motor activity in the morning, while males were more active at night. These findings, consistent across different ages and genetic backgrounds in mice, further reinforce the robustness of these observations.^( 25 )^

Motor assessments are widely used to monitor motor behavior, particularly in longitudinal analyses. Herein, we conducted this assessment weekly but did not identify any significant differences in the evaluated measures or habituation patterns. These results suggest that the adopted temporal interval may be appropriate for supporting the maintenance of continuous behavior without inducing habituation-related stress or demotivation.

The novel object recognition test showed significant differences in the effects of time on the RI and average speed parameters. Notably, a higher RI was observed in the morning, which can be explained by the rats’ greater motivation to gather information about their environment. Additionally, the results showed a greater speed in the afternoon. This finding supports the theory of greater activity during the dark phase, as reported by Nelson et al. and Takahashi et al.^( 15 , 26 )^ Regarding the type of exploration, the novel object was significantly more explored than the familiar object, especially by females in the morning. This pattern was also observed in the control group of another study, although the differences were not statistically significant.^( 27 )^ In contrast, the effect of sex did not show significant differences in any of the evaluated parameters, which aligns with the findings of most of the articles included in a review.^( 28 )^

In the Morris water maze test, the latency to find the platform did not vary significantly with respect to sex or time of day. However, females took longer to locate the platform than males in the afternoon. Zorzo et al.^( 29 )^ also found no significant differences in latency between genders, although females exhibited higher latency values than males. The same study evaluated the speed at which the platform was found, and the results corroborated our findings, showing that females were faster than males, especially in the afternoon. However, our study found that the speed parameter was significantly affected by time, with both sexes showing greater speed in the morning. This suggests that the motivation to escape from the water, an aversive stimulus, may be more pronounced during the alert phase of rodents, as discussed by Valentinuzzi et al.^( 30 )^ For this test (herein), there was a careful analysis that led to a reduction in the final statistics by removing the two animals that did not find the platform throughout the 60 s period of the test.

For the spatial evaluation of the navigation strategy by the Morris water maze test by quadrant, a significant difference was observed in the time spent in each quadrant, particularly in the one containing the platform (SE) compared to the others. Additionally, there was a greater exploration of the initial quadrant during the test than of the other quadrants. This pattern has also been reported previously.^( 29 , 31 )^ Although not significant, males found the platform faster than females and had more navigation strategies, showing a longer time spent in the target quadrant, than females, a result also reported by other studies.^( 29 , 32 )^

The time of day in behavioral assessments of rodents is a relevant biological factor that is often neglected in preclinical studies.^( 15 )^ Variations in the timing of behavioral test applications can significantly influence results, as even during the light phase, animal behavior can vary between the morning and afternoon, as observed in some parameters of our study. Furthermore, sex was identified as the most relevant biological factor associated with behavioral variation.

Our results reinforce the importance of understanding which behavioral aspects are most affected by the time of day, in addition to the impact of the circadian cycle and sex in experimental designs. This understanding is essential for adjusting experimental protocols according to the most relevant variables. The lack of standardization in test timings may contribute to inconsistencies between studies, as pointed out by Nelson et al.^( 15 )^ and Valentinuzzi et al.^( 30 )^ Experimental strategies, such as reversing the light cycle, can align test timings in the active phases of animals, ensuring greater accuracy in results and reproducibility of behavioral research.

These factors highlight the need for a careful approach when planning experiments with rodents, considering not only the environment and diet, but also the intrinsic characteristics of the animals, such as nocturnal behavior and sensitivity to timing. Understanding these aspects can significantly improve the quality and reliability of data obtained in preclinical studies, especially in models of neurodegenerative and psychiatric disorders as well as in investigations of neurotoxic effects.

## CONCLUSION

This study highlights the importance of considering factors, such as biological sex and time of day, in preclinical behavioral studies. These variables significantly influence locomotor, cognitive, and memory aspects in rodents, emphasizing the need for more meticulous experimental planning. Incorporating analyses that account for circadian rhythm differences and sex-specific characteristics not only enhances the robustness of preclinical models but also contributes to reducing variability and improving the reliability of behavioral data. By adopting these approaches, future research can generate more consistent and interpretable findings, ultimately strengthening the quality of experimental designs in behavioral neuroscience.
